# Safety, efficacy and pharmacokinetics of BPI-9016M in c-MET overexpression or *MET* exon 14 skipping mutation patients with locally advanced or metastatic non-small-cell lung cancer: a phase Ib study

**DOI:** 10.1186/s12885-022-10500-y

**Published:** 2023-04-11

**Authors:** Xingsheng Hu, Xinge Cui, Ziping Wang, Yunpeng Liu, Ying Luo, Wei Zhong, Hui Zhao, Mengxing Yao, Da Jiang, Mingxia Wang, Minjiang Chen, Xin Zheng, Lieming Ding, Yang Wang, Xiaobin Yuan, Pengxiang Wu, Bei Hu, Xiaohong Han, Yuankai Shi

**Affiliations:** 1 Department of Medical Oncology, National Cancer Center/National Clinical Research Center for Cancer/Cancer Hospital, Chinese Academy of Medical Sciences & Peking Union Medical College, Beijing Key Laboratory of Clinical Study on Anticancer Molecular Targeted Drugs, No. 17 Panjiayuan Nanli, Chaoyang District, Beijing, 100021 China; 2 Clinical Pharmacology Research Center, State Key Laboratory of Complex Severe and Rare Diseases, NMPA Key Laboratory for Clinical Research and Evaluation of Drug, Beijing Key Laboratory of Clinical PK & PD Investigation for Innovative Drugs, Peking Union Medical College Hospital, Chinese Academy of Medical Sciences & Peking Union Medical College, No.1 Shuaifuyuan, Dongcheng District, Beijing, 100730 China; 3 Department of Thoracic Oncology, Beijing Cancer Hospital, Beijing, China; 4 Department of Medical Oncology, The First Hospital of China Medical University, Liaoning, China; 5 Department of Respiratory and Critical Care Medicine, Peking Union Medical College Hospital, Chinese Academy of Medical Sciences & Peking Union Medical College, Beijing, China; 6 Department of Respiratory Medicine, The Second Affiliated Hospital of Anhui Medical University, Anhui, China; 7 Department of Oncology, Hebei Tumor Hospital, Hebi, China; 8 Betta Pharmaceuticals Co., Ltd, Hangzhou, China

**Keywords:** BPI-9016M, Mesenchymal-epithelial transition factor, Non-small-cell lung cancer, Safety, Efficacy, Pharmacokinetics

## Abstract

**Background:**

As a potential target receptor tyrosine kinase, mesenchymal-epithelial transition factor (*MET*) exhibits high aberrant expression across various tumors. This study aimed to evaluated the safety, tolerability, efficacy and pharmacokinetics (PK) of BPI-9016M, a novel tyrosine kinase inhibitor (TKI) targeting c-MET, in c-MET overexpression or *MET* exon 14 skipping mutation patients with locally advanced or metastatic non-small-cell lung cancer (NSCLC).

**Methods/design:**

In this two-part multicenter phase Ib study, eligible patients with locally advanced or metastatic NSCLC harboring c-MET overexpression or *MET* exon 14 skipping mutation were enrolled into Part A (tested positive for c-MET overexpression [immunohistochemical staining score ≥ 2+]; 300 mg quaque die [QD], 450 mg QD and 600 mg QD cohorts) or Part B (tested positive for *MET* exon 14 skipping mutation; 400 mg bis in die [BID] cohort), respectively. The primary endpoints were safety, objective response rate (ORR) and disease control rate (DCR), the second endpoints were PK parameters, progression-free survival (PFS) and overall survival (OS).

**Results:**

Between March 15, 2017 and September 18, 2021, 38 patients were enrolled (Part A, *n* = 34; Part B, *n* = 4). Of 38 patients, 32 (84.2%) patients completed the treatment protocol. As of the data cut-off date on January 27, 2022, all patients reported at least one treatment-emergent adverse event (TEAE). Ninety-two point one percent (35/38) of patients experienced treatment-related adverse events (TRAEs), and grade ≥ 3 TRAEs were observed in 11 (28.9%) patients. The most common TRAEs were elevated alanine aminotransferase (ALT, 14/38, 36.8%) and elevated aspartate aminotransferase (AST, 11/38, 28.9%). Only one (2.6%) patient had treatment-related serious adverse event (SAE) in 600 mg QD cohort due to thrombocytopenia. PK analysis showed BPI-9016M and its main metabolites (M1 and M2-2) reached steady state after seven days of continuous administration. At the dose of 300 mg QD and 450 mg QD, the exposure of BPI-9016M increased with increasing dose. Exposure of BPI-9016M was similar at 450 mg QD and 600 mg QD, which may exhibit a saturation trend. In all patients, ORR and DCR were 2.6% (1/38, 95% confidence interval [CI] 0.1–13.8%) and 42.1% (16/38, 95% CI 26.3–59.2%), respectively. Only one partial response (PR) patient was observed at a dose of 600 mg QD in Part A. In Part B, DCR was 75.0% (3/4, 95% CI 19.4–99.4%). The median PFS and OS in all 38 patients were 1.9 months (95% CI 1.9–3.7) and 10.3 months (95% CI 7.3–not evaluable [NE]), respectively.

**Conclusion:**

BPI-9016M showed manageable safety profile in c-MET overexpression or *MET* exon 14 skipping mutation patients with locally advanced or metastatic NSCLC, but showed limited efficacy.

**Trial registration:**

Clinicaltrials.gov NCT02929290 (11/10/2016).

**Supplementary Information:**

The online version contains supplementary material available at 10.1186/s12885-022-10500-y.

## Background

C-mesenchymal-epithelial transition factor (c-MET) is a transmembrane receptor with autonomic phosphorylation activity encoded by *MET*. Normal c-MET pathway promotes tissue differentiation and repair, while c-MET dysregulation can result in tumor cell proliferation and metastasis [1]. *MET* amplification, *MET* exon 14 skipping mutation and c-MET overexpression are main mechanisms inducing aberrant activation of c-MET pathway in lung cancer. *MET* amplification has been reported in 5–26% of patients with non-small-cell lung cancer (NSCLC) after resistance to epidermal growth factor receptor (EGFR) inhibitor [2–6], with *MET* exon 14 skipping mutation in approximately 3% of NSCLC [7–9], and c-MET overexpression in about 35–72% of NSCLC [10, 11]. It appears that overexpression of c-MET leads to activation of downstream signaling pathways via ligand-independent phosphorylation, which is associated with poorer outcomes in patients with NSCLC [11]. Overall, c-MET receptor has become a new trend in the research field of targeted therapy for NSCLC.

Currently, several phase I and phase II studies have demonstrated the promising activity of MET tyrosine kinase inhibitors (TKIs), such as tepotinib, capmatinib and savolitinib, in NSCLC patients with c-MET dysregulation [12]. In a prospective single-arm phase II (VISION) study, tepotinib had an objective response rate (ORR) of 46% and a median duration of response (DoR) of 11.1 months in NSCLC patients with *MET* exon 14 skipping mutation [13], which was approved by the Pharmaceuticals and Medical Devices Agency of Japan on March 25, 2020. Subsequently, capmatinib [14] was approved by the U.S Food and Drug Administration on May 6, 2020 and savolitinib [15] was approved by the National Medical Products Administration of China on June 22, 2021 due to their promising clinical efficacy (ORR were 41% and 42.9% for capmatinib and savolitinib, respectively) in patients harboring *MET* exon 14 skipping mutation. However, the indication of these targeted drugs mostly focus on patients with *MET* exon 14 skipping mutation, primary *MET* amplification or secondary amplification after resistance to EGFR TKIs [13–16]. In the phase I study of capmatinib in patients with *MET*-positive solid tumors, 11 of 76 patients showed c-MET immunohistochemical (IHC) score 3 + , the disease control rate (DCR) in all patients were 26% [17]. Phase I study of telisotuzumab vedotin, an antibody-drug conjugate targeting c-MET, enrolled 48 patients with advanced solid tumors with c-MET H-score of ≥ 150. Among the 16 patients with NSCLC, the DCR was 56% (95% confidence interval [CI] 3.0–80.2%) [18]. However, studies of MET TKIs in NSCLC patients with c-MET overexpression remains limited.

BPI-9016M is a novel small-molecule TKI targeting both c-MET and AXL developed by Betta Pharmaceuticals Co., Ltd, Hangzhou, China., which can suppress tumor cell growth, migration and invasion of lung adenocarcinoma [19]. Previous first-in-human phase I dose-escalation study of BPI-9016M in Chinese patients with advanced NSCLC demonstrated its favorable safety, tolerability and pharmacokinetics (PK) profiles. No dose-limiting toxicity (DLT) occurred even at a dose of 800 mg quaque die (QD). The most common toxicities were alanine aminotransferase (ALT) elevation, bilirubin increased and dysgeusia [20]. Thus, we intended to further evaluate the safety, tolerability, efficacy and PK profile of different doses of BPI-9016M in patients with locally advanced or metastatic NSCLC harboring c-MET overexpression or *MET* exon 14 skipping mutation.

## Methods/design

This was a multicenter, single-arm, open-label, phase Ib expansion cohort study conducted at seven hospitals in China. The study was approved by the ethics committee of each participating hospital. Written informed consent was obtained from each patient. This study was registered with clinicalTrials.gov, NCT02929290.

### Patients

The included patients were divided into Part A (c-MET overexpression) and Part B (*MET* exon 14 skipping mutation) according to c-MET/*MET* alteration status. Patients were eligible if they fulfilled the following criteria: 1) aged ≥ 18 years; 2) histologically or cytologically confirmed with locally advanced or metastatic (stage IIIB/IIIC/IV) NSCLC including lung sarcomatoid carcinoma, for which surgery or radiotherapy was unsuitable, the stage was determined by the American Joint Committee on Cancer (AJCC) 7 th edition staging system; 3) c-MET overexpression (IHC staining score ≥ 2 +) or *MET* exon 14 skipping mutation (blood and tissue samples were collected to perform next generation sequencing [NGS]); 4) an Eastern Cooperative Oncology Group (ECOG) performance status (PS) of 0–1; 5) a life expectancy exceeding 12 weeks; 6) at least one measurable lesion; 7) adequate hematological, hepatic and renal function, mainly including bone marrow function: absolute neutrophil count (ANC) ≥ 1.5 × 10^9^/L, platelets ≥ 100 × 10^9^/L and hemoglobin ≥ 9 g/dL; coagulation function: international normalized ratio of prothrombin time or partial thromboplastin time < 1.5 times the upper limit of normal range (ULN); liver function: total serum bilirubin ≤ 1.5 times ULN, aspartate aminotransferase (AST) and ALT ≤ 2.5 times ULN (AST and ALT ≤ 5 times ULN are allowed if liver metastases are present); renal function: serum creatinine ≤ 1.5 times ULN or endogenous creatinine clearance ≥ 60 mL/min (calculated using the Cockcroft-Gault formula); 8) patients should not have received any cytotoxic chemotherapy, radiotherapy, immunotherapy or hormone therapy within four weeks before BPI-9016M treatment; 9) prior small-molecule targeted therapies should have been terminated more than 14 days or five half-lives of the drug (whichever was longer) before BPI-9016M treatment; 10) all treatment-related adverse events (TRAEs) (except for alopecia) should have resolved to grade ≤ 1.

The key exclusion criteria were 1) any other malignancies within five years (except for cured cervical cancer in situ, skin squamous cell carcinoma or papillary thyroid cancer); 2) prior treatment with other hepatocyte growth factor (HGF)/c-MET small-molecule inhibitors or HGF/c-MET monoclonal antibodies, including cabozantinib, merestinib, glesatinib, emibetuzumab, ficlatuzumab, etc.; 3) confirmed anaplastic lymphoma kinase (*ALK*), c-ros oncogene 1 (*ROS1*) rearrangement or ALK positive detected by Ventana IHC ALK (D5F3); 4) symptomatic brain metastasis; 5) thrombus or high risk of developing thrombus; 6) any situations influencing the swallowing of BPI-9016M, or seriously influencing the absorption of BPI-9016M or PK parameters.

### Study design

Patients enrolled in this study were divided into two parts. In Part A, patients were enrolled into the 300 mg QD, 450 mg QD and 600 mg QD dose cohorts, the tumor samples collected during the pre-screening period were detected by IHC staining, and IHC staining score was detected as ≥ 2 + could be enrolled. Patients with pre-existing c-MET overexpression at hospital diagnosis were also included in Part A. In Part B, patients in the 400 mg bis in die (BID) dose cohort, blood and tissue samples were collected to perform NGS to determine the *MET* exon 14 skipping mutation. Patients harboring pre-existing *MET* exon 14 skipping mutation at hospital diagnosis were also enrolled into Part B. In Part A, three dose cohorts were designed for locally advanced or metastatic (stage IIIB/IIIC/IV) NSCLC patients with c-MET overexpression based on the results from previous phase I dose escalation study [20]. BPI-9016M was given orally once a day until disease progression or intolerable toxicity, and 28 days were deemed as a cycle. The starting dose cohort of BPI-9016M was 300 mg QD. If three or more of 12 patients had disease control (complete response [CR], partial response [PR] or stable disease [SD]) at week 16, this cohort could be expanded to enroll more patients. If less than three of 12 patients had disease control, the next dose cohort would begin. Combined with previous PK and efficacy results [20], dose at > 600 mg QD would not be evaluated considering the limited additional antitumor activity for patients with c-MET overexpression.

Based on a previous population PK model analysis (data unpublished), following 400 mg BID, area under the concentration-time curve (AUC) values for both BPI-9016M and its main plasma metabolites were comparable with that at 800 mg QD, and trough concentration relatively elevated whereas maximum concentration (C_max_) was reduced. These data suggest 400 mg BID may better maintains efficacy and decreases the overall incidence and severity of TRAEs. Thus, in Part B, a dose of 400 mg BID was selected for patients with locally advanced or metastatic NSCLC who had *MET* exon 14 skipping mutation.

Imaging examinations including computed tomography (CT), cranial contrast-enhanced CT or magnetic resonance imaging were performed every eight weeks at the first 12 cycles, and every 12 weeks thereafter. For patients with disease progression, they could continue the BPI-9016M treatment if the investigator and sponsor assessed that BPI-9016M could still bring benefit. Dose adjustment was allowed according to the severity of toxicity [Supplementary Table 1]. BPI-9016M should be interrupted when patients had suspected interstitial lung disease (ILD), and should be discontinued when ILD was confirmed. If patients benefited from BPI-9016M without grade ≥ 3 TRAEs and < 33% of patients experienced DLT in the dose cohort, they could enter a higher dose cohort to continue the BPI-9016M treatment after agreement among the investigator, sponsor and patients. In this situation, these patients would be included in the higher dose cohort for efficacy analysis.

### Endpoints and assessment

The primary endpoints were safety, ORR and DCR assessed by investigators. Secondary endpoints included progression-free survival (PFS) and overall survival (OS). Adverse events (AEs) were recorded during the study period and until 28 days after the last dose of BPI-9016M, and graded according to the National Cancer Institute Common Terminology Criteria for Adverse Events (CTCAE) version 4.03. AEs referred to treatment-emergent adverse events (TEAEs) and TRAEs were assessed by investigators. Serious adverse events (SAEs) referred to AEs meeting at least one of the following criteria: event leading to death; life-threatening event; event requiring hospitalization or extension of hospital stay; event leading to persistent or evident disability or dysfunction; event leading to congenital malformation or birth defect or event which might lead to injury or that need medical treatment to avoid the above mentioned important medical events. Response was evaluated according to the Response Evaluation Criteria In Solid Tumors (RECIST) version 1.1 [21] by investigators.

Efficacy was analyzed in full analysis set (FAS) which included patients with at least one dose of BPI-9016M. The safety was analyzed in safety set (SS) which included patients with at least one dose of BPI-9016M with safety assessment. ORR was defined as the proportion of patients with CR or PR. DCR was defined as the proportion of patients with CR, PR or SD. PFS was defined as the time from the initiation of BPI-9016M treatment to radiographic progression or death of any cause, whichever occurred first. OS was defined as the time from the initiation of BPI-9016M treatment to death of any cause.

### PK assessment

PK profiles of BPI-9016M and its metabolites (M1 and M2-2) in different dose cohorts (300 mg QD, 450 mg QD and 600 mg QD) were evaluated during the continuous administration of BPI-9016M for 28 days in Part A which is also the pharmacokinetics analysis set (PKAS). Serial peripheral blood samples were collected at pre-dose on Cycle 1 Day 8, Cycle 1 Day 15 and Cycle 1 Day 22 for the trough concentration (C_trough_) obtained during the dosing interval. Serial peripheral blood samples were collected at Cycle 1 Day 28 of BPI-9016M treatment and at consecutive three days after Cycle 1 without BPI-9016M treatment at 648 h, 649 h, 650 h, 652 h, 654 h, 656 h, 660 h, 672 h, 696 h, 720 h post-dose for PK analysis. After Cycle 1, the C_trough_ was measured every 4 or 8 or 12 weeks. The whole blood (3 mL) was taken intravenously and placed in sodium heparin tubes, gently turned over 15 times, and centrifuged within 1 h (4 °C 3000 rpm for 10 min). Two portions of the centrifuged upper plasma should be transferred to 1.5 mL centrifuge tubes with identification labels and stored at -80 °C. The following PK parameters were assessed: time to maximum plasma concentration at steady state (T_max,ss_), overall body clearance at steady state for extravascular dosage (CL_ss_/F), terminal half-life (T_1/2_), plasma C_trough_ observed in Cycle 1 at steady state, total volume of drug distribution at steady state according to the terminal phase (V_z,ss_/F), AUC over a dosing interval (AUC_tau_), ratio of metabolites to parent drug calculated based on AUC_tau_ (MP ratio).

### Statistical analysis

Statistical analyses were performed using SAS version 9.4 (SAS Institute, Cary, NC, USA). Age was expressed as median (range), and categorical variables were expressed as frequency (percentage). The 95% CIs of ORR and DCR were calculated using Clopper-Pearson method. The 95% CIs of PFS and OS were calculated using Brookmeyer-Crowley method. PK parameters were expressed as mean (standard deviation) or median (range), and analyzed using Phoenix version 8.0 (Certara, LP, Princeton, NJ, USA). No imputation method was performed for missing data.

## Results

### Patient characteristics

**Fig. 1:**
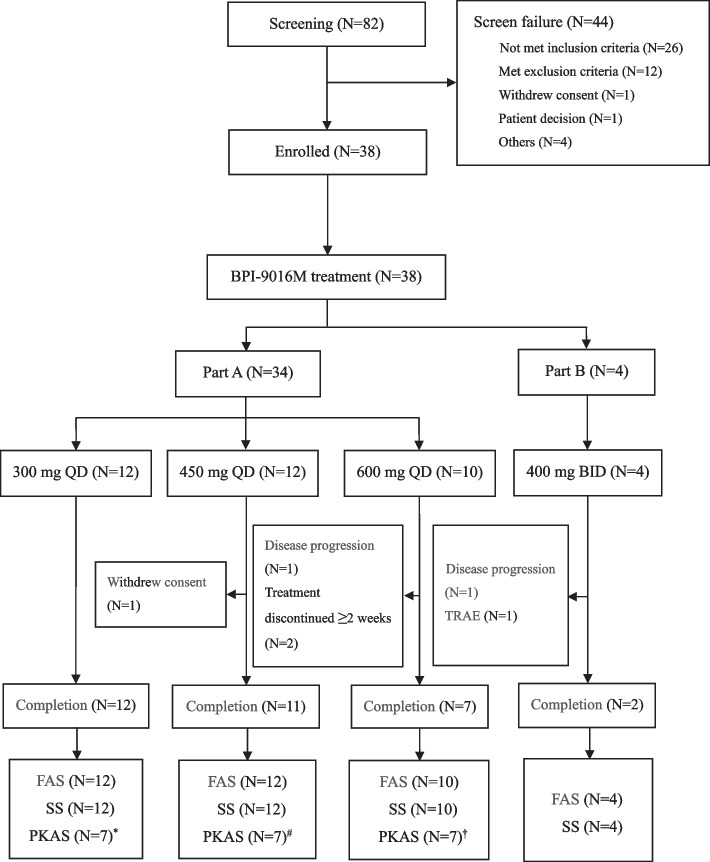
Flow chart. ^*^In the 300 mg QD cohort, one patient had protocol violation and no exact administration time of BPI-9016M, the sparse PK data of four patients were analyzed based on population PK analysis without PK modeling, which were not included in PKAS. ^#^In the 450 mg QD cohort, the sparse PK data of five patients were analyzed based on population PK analysis without PK modeling, which were not included in PKAS. ^†^In the 600 mg QD cohort, one patient discontinued over two-week treatment of BPI-9016M due to the poor compliance, the sparse PK data of two patients were analyzed based on population PK analysis without PK modeling, which were not included in PKAS *QD* quaque die, *BID* bis in die, *TRAE* treatment-related adverse event, *FAS* full analysis set, *SS* safety set, *PKAS* pharmacokinetics analysis set, *PK* pharmacokinetics

Between March 15, 2017 and September 18, 2021, 82 patients were screened, 44 patients failed screened, and all the 38 patients were included in FAS and SS (Fig. 1). In Part A, 34 patients with c-MET overexpression were enrolled (12 patients in 300 mg QD cohort, 12 patients in 450 mg QD cohort and 10 patients in 600 mg QD cohort). In Part B, four patients with *MET* exon 14 skipping mutation were enrolled into 400 mg BID cohort. Of these patients, the median age was 60.0 years (range, 47–78 years), with 20 (52.6%) patients being male. Most of them had an ECOG PS of 1 (76.3%), lung adenocarcinoma (94.7%), stage IV disease (92.1%), and had been treated with prior chemotherapy or targeted therapy (97.4%; Table 1).

**Table 1:** Baseline characteristics of BPI-9016M treated patients

Characteristics	Part A			Part B	Total (*n* = 38)
	**300 mg QD (*n* = 12)**	**450 mg QD (*n* = 12)**	**600 mg QD (*n* = 10)**	**400 mg BID (*n* = 4)**	
**Age (years), median (range)**	60.0 (47–78)	61.5 (50–66)	57.0 (52–65)	67.0 (67–76)	60.0 (47–78)
**Sex, n (%)**					
Male	8 (66.7)	4 (33.3)	7 (70.0)	1 (25.0)	20 (52.6)
Female	4 (33.3)	8 (66.7)	3 (30.0)	3 (75.0)	18 (47.4)
**ECOG PS, n (%)**					
0	4 (33.3)	3 (25.0)	2 (20.0)	0	9 (23.7)
1	8 (66.7)	9 (75.0)	8 (80.0)	4 (100.0)	29 (76.3)
**Tumor histology, n (%)**					
Adenocarcinoma	11 (91.7)	12 (100.0)	9 (90.0)	4 (100.0)	36 (94.7)
Squamous cell carcinoma	0	0	1 (10.0)	0	1 (2.6)
Poorly-differentiated carcinoma	1 (8.3)	0	0	0	1 (2.6)
**Stage, n (%)**					
IIIB	1 (8.3)	1 (8.3)	1 (10.0)	0	3 (7.9)
IV	11 (91.7)	11 (91.7)	9 (90.0)	4 (100.0)	35 (92.1)
**c-MET/*MET* alteration status, n (%)**					
c-MET overexpression	12 (100.0)	12 (100.0)	10 (100.0)	NE	34 (89.5)
*MET* exon 14 skipping mutation	NE	NE	NE	4 (100.0)	4 (10.5)
**Number of metastatic lesions, n (%)**					
< 3	5 (41.7)	5 (41.7)	4 (40.0)	2 (50.0)	16 (42.1)
≥ 3	7 (58.3)	7 (58.3)	6 (60.0)	2 (50.0)	22 (57.9)
**Prior treatment, n (%)**					
Surgery	1 (8.3)	4 (33.3)	4 (40.0)	1 (25.0)	10 (26.3)
Radiotherapy	4 (33.3)	4 (33.3)	0	1 (25.0)	9 (23.7)
Chemotherapy or targeted therapy	12 (100.0)	12 (100.0)	10 (100.0)	3 (75.0)	37 (97.4)
Others	1 (8.3)	0	0	0	1 (2.6)
**Prior lines of systemic therapy, n (%)**					
0	0	0	0	1 (25.0)	1 (2.6)
1	3 (25.0)	2 (16.7)	1 (10.0)	0	6 (15.8)
2	5 (41.7)	3 (25.0)	5 (50.0)	2 (50.0)	15 (39.5)
3	2 (16.7)	3 (25.0)	2 (20.0)	0	7 (18.4)
4	2 (16.7)	3 (25.0)	1 (10.0)	1 (25.0)	7 (18.4)
≥ 5	0	1 (8.3)	1 (10.0)	0	2 (5.3)

Data are n (%), unless otherwise stated

*ECOG* Eastern Cooperative Oncology Group, *PS* performance status, *MET* mesenchymal-epithelial transition factor, *QD* quaque die, *BID* bis in die, *NE* not evaluable

### Safety

As of the data cut-off date on January 27, 2022, the median duration of BPI-9016M exposure was 2.1 months (range, 0.3–9.3). All the 38 patients were included in SS and had at least one TEAE, and grade ≥ 3 TEAEs were reported in 14 (36.8%) patients. Five (13.2%) patients had SAEs. One patient in the 450 mg QD cohort suffered TEAE of pulmonary infection leading to death, which was deemed unrelated to BPI-9016M.

A total of 35 (92.1%) patients had TRAEs, and grade ≥ 3 TRAEs occurred in 11 (28.9%) patients. One (2.6%) patient had TRAE (grade 3 palmar-plantar erythrodysesthesia [PPE]) leading to treatment discontinuation in 400 mg BID cohort. One (2.6%) patient had treatment-related SAE in 600 mg QD cohort (patient personally discontinued drug due to thrombocytopenia, which recovered spontaneously). The most common TRAEs were elevated ALT (14/38, 36.8%), elevated AST (11/38, 28.9%), dysgeusia (9/38, 23.7%), PPE (9/38, 23.7%) and constipation (8/38, 21.1%). The most common grade ≥ 3 TRAE was PPE (4/38, 10.5%; Table 2). All TRAEs observed in this study were listed in Supplementary Table 2.

**Table 2:** ≥ 10% TRAEs of BPI-9016M in SS

TRAEs	Part A						Part B		Total (*n* = 38) *n* (%)	
	**300 mg QD (*n* = 12) n (%)**		**450 mg QD (*n* = 12) n (%)**		**600 mg QD (*n* = 10) n (%)**		**400 mg BID (*n* = 4) n (%)**			
	**Any grade**	**Grade 3–4**	**Any grade**	**Grade 3–4**	**Any grade**	**Grade 3–4**	**Any grade**	**Grade 3–4**	**Any grade**	**Grade 3–4**
Elevated ALT	3 (25.0)	0	5 (41.7)	0	3 (30.0)	0	3 (75.0)	0	14 (36.8)	0
Elevated AST	2 (16.7)	0	5 (41.7)	0	1 (10.0)	0	3 (75.0)	0	11 (28.9)	0
Dysgeusia	1 (8.3)	0	3 (25.0)	0	5 (50.0)	1 (10.0)	0	0	9 (23.7)	1 (2.6)
PPE	1 (8.3)	0	3 (25.0)	1 (8.3)	1 (10.0)	0	4 (100.0)	3 (75.0)	9 (23.7)	4 (10.5)
Constipation	2 (16.7)	0	5 (41.7)	0	1 (10.0)	0	0	0	8 (21.1)	0
Elevated blood creatinine	3 (25.0)	0	2 (16.7)	0	1 (10.0)	0	1 (25.0)	0	7 (18.4)	0
Decreased Ccr	2 (16.7)	0	2 (16.7)	0	2 (20.0)	0	0	0	6 (15.8)	0
Asthenia	0	0	2 (16.7)	0	3 (30.0)	1 (10.0)	0	0	5 (13.2)	1 (2.6)
Decreased appetite	2 (16.7)	0	2 (16.7)	0	1 (10.0)	1 (10.0)	0	0	5 (13.2)	1 (2.6)
Proteinuria	0	0	3 (25.0)	1 (8.3)	1 (10.0)	0	0	0	4 (10.5)	1 (2.6)
Hypertension	1 (8.3)	0	0	0	2 (20.0)	1 (10.0)	1 (25.0)	0	4 (10.5)	1 (2.6)

Data are n (%). One patient in the 450 mg QD cohort suffered adverse event of pulmonary infection leading to death, which was deemed unrelated to BPI-9016M

*TRAEs* treatment-related adverse events, *SS* safety set, *ALT* alanine aminotransferase, *AST* aspartate aminotransferase, *PPE* palmar-plantar erythrodysesthesia, *Ccr* creatinine clearance, *QD* quaque die, *BID* bis in die

### Efficacy

**Fig. 2:**
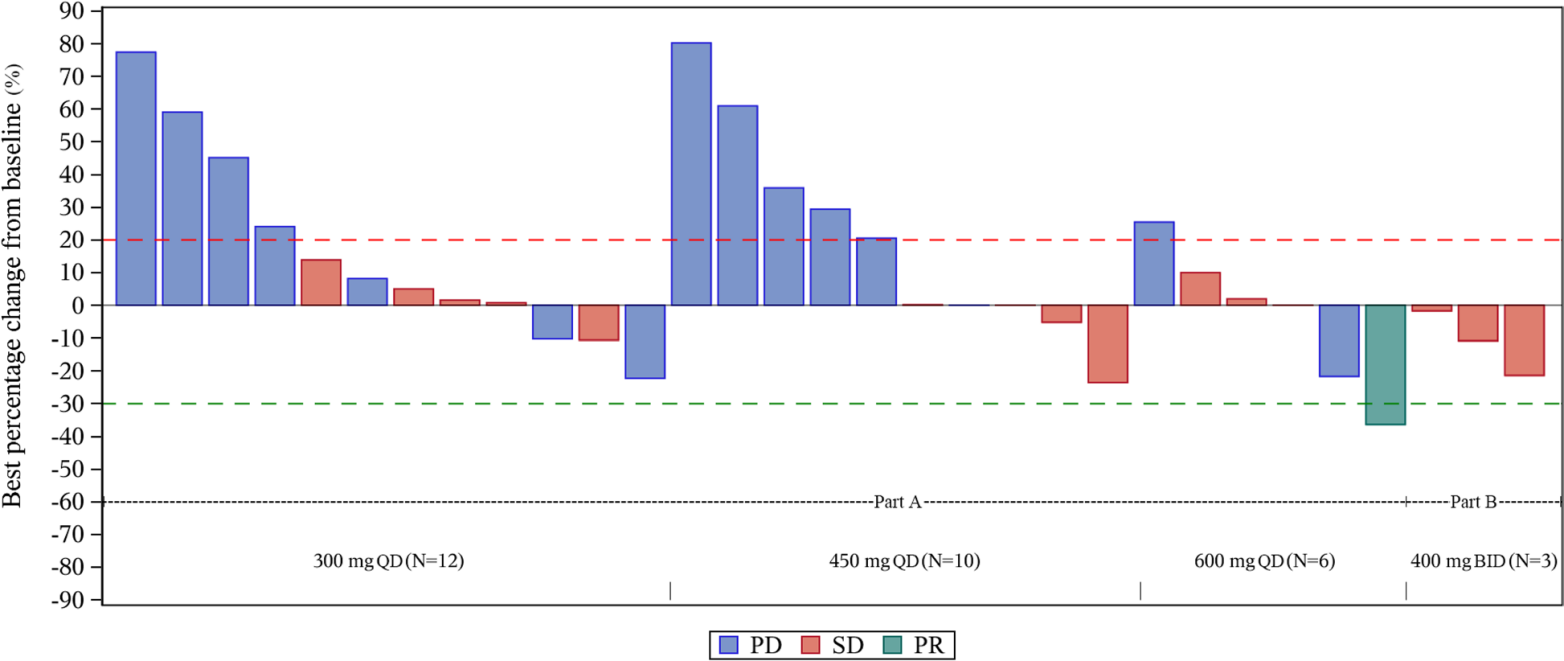
Waterfall plots of best change in the target lesion size from baseline of BPI-9016M treated patients. The dashed lines at 20% and -30% indicate the thresholds for PD and PR, respectively. Seven patients (two patients in the 450 mg QD cohort, four patients in the 600 mg QD cohort and one patient in the 400 mg BID cohort) were not evaluable *QD* quaque die, *BID* bis in die, *PD* progressive disease, *SD* stable disease, *PR* partial response

Overall, 38 patients were included in FAS for efficacy analysis (Table 3, Fig. 2). The ORR was 2.6% (1/38, 95% CI 0.1–13.8%), and the DCR was 42.1% (16/38, 95% CI 26.3–59.2%). One (2.6%) patient achieved PR which was in 600 mg QD cohort in Part A, and 15 (39.5%) of all patients achieved SD. In Part A, the DCR of the 300 mg QD, 450 mg QD and 600 mg QD cohort were 41.7% (5/12, 95% CI 15.2–72.3%), 33.3% (4/12, 95% CI 9.9–65.1%) and 40.0% (4/10, 95% CI 12.2–73.8%), respectively. In Part B, three patients achieved SD, and DCR in 400 mg BID cohort was 75.0% (3/4, 95% CI 19.4–99.4%).

**Table 3:** Efficacy of BPI-9016M in FAS

BOR, n (%)	Part A			Part B	Total (*n* = 38) n (%)
	**300 mg QD (*n* = 12)**	**450 mg QD (*n* = 12)**	**600 mg QD (*n* = 10)**	**400 mg BID (*n* = 4)**	
CR	0	0	0	0	0
PR	0	0	1 (10.0)	0	1 (2.6)
SD	5 (41.7)	4 (33.3)	3 (30.0)	3 (75.0)	15 (39.5)
PD	7 (58.3)	6 (50.0)	2 (20.0)	0	15 (39.5)
NE	0	2 (16.7)	4 (40.0)	1 (25.0)	7 (18.4)
ORR (95% CI)	0 (0, 26.5)	0 (0, 26.5)	10.0 (0.3, 44.5)	0 (0, 60.2)	2.6 (0.1, 13.8)
DCR (95% CI)	41.7 (15.2, 72.3)	33.3 (9.9, 65.1)	40.0 (12.2, 73.8)	75.0 (19.4, 99.4)	42.1 (26.3, 59.2)
PFS, months					
Median (95% CI)	1.9 (1.9, 5.6)	1.9 (1.1, 3.8)	1.8 (1.2, 3.9)	3.4 (3.2, 3.7)	1.9 (1.9, 3.7)
OS, months					
Median (95% CI)	NE (4.0, NE)	9.3 (2.6, NE)	NE (2.6, NE)	NE (NE, NE)	10.3 (7.3, NE)

*FAS* full analysis set, *BOR* best of response, *CR* complete response, *PR* partial response, *SD* stable disease, *PD* progressive disease, *NE* not evaluable, *ORR* objective response rate, *CI* confidence interval, *DCR* disease control rate, *PFS* progression-free survival, *OS* overall survival, *QD* quaque die, *BID* bis in die

**Fig. 3:**
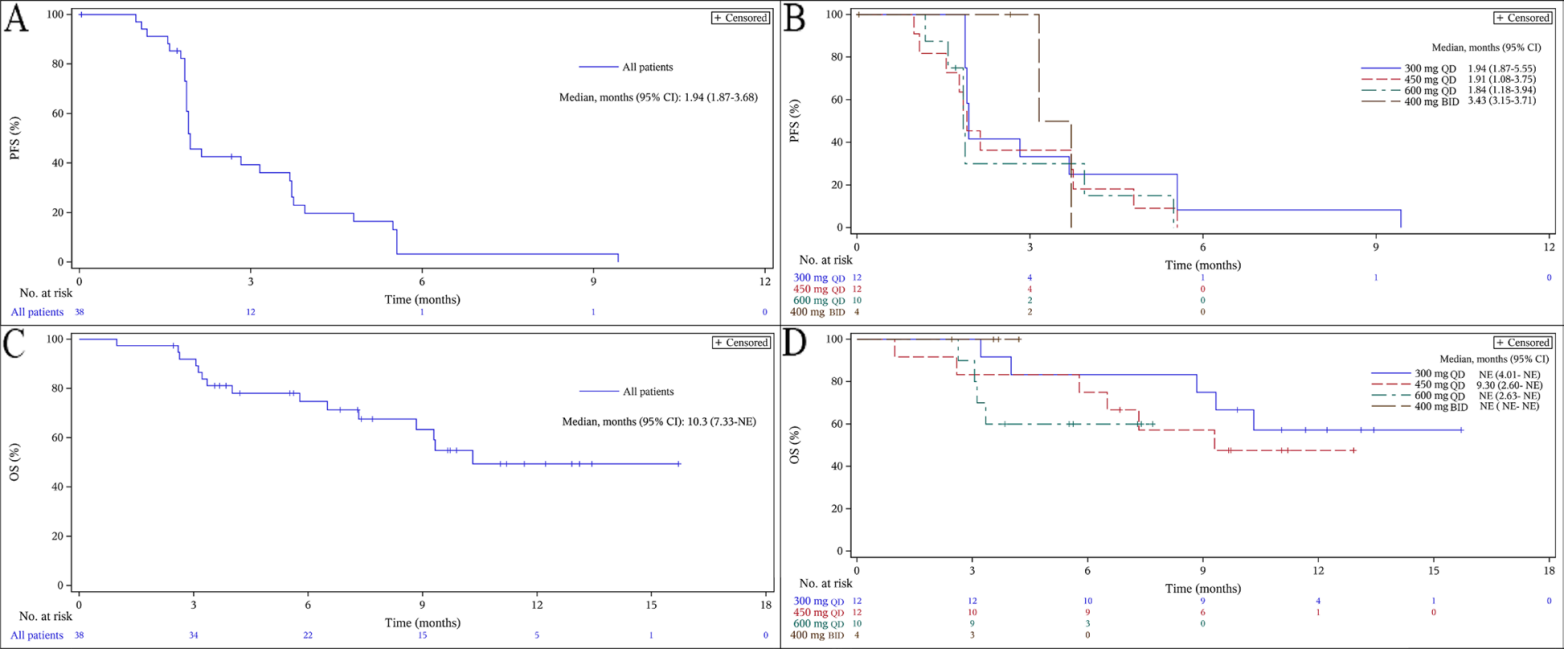
Kaplan–Meier curves for PFS and OS of BPI-9016M treated patients. **A:** Kaplan–Meier curve of PFS for all patients (*N* = 38). **B:** Kaplan–Meier curve of PFS for all four dose cohorts (300 mg QD, 450 mg QD, 600 mg QD and 400 mg BID). **C:** Kaplan–Meier curve of OS for all patients (*N* = 38). **D:** Kaplan–Meier curve of OS for all four dose cohorts (300 mg QD, 450 mg QD, 600 mg QD and 400 mg BID) *PFS* progression-free survival, *OS* overall survival, *QD* quaque die, *BID* bis in die, *CI* confidence interval, *NE* not evaluable

The median PFS in all 38 patients was 1.9 months (95% CI 1.9–3.7; Fig. 3A). In Part A, the median PFS in the 300 mg QD, 450 mg QD and 600 mg QD cohort was 1.9 months (95% CI 1.9–5.6), 1.9 months (95% CI 1.1–3.8) and 1.8 months (95% CI 1.2–3.9), respectively. In Part B, the median PFS was 3.4 months (95% CI 3.2–3.7) (Fig. 3B). The median OS in all patients was 10.3 months (95% CI 7.3–not evaluable [NE]; Fig. 3C). In Part A, the median OS was NE (95% CI 4.0 months–NE), 9.3 months (95% CI 2.6–NE) and NE (95% CI 2.6 months–NE) in the 300 mg QD, 450 mg QD and 600 mg QD cohort, respectively. In Part B, the median OS was NE (95% CI NE–NE) (Fig. 3D).

### PK

**Fig. 4:**
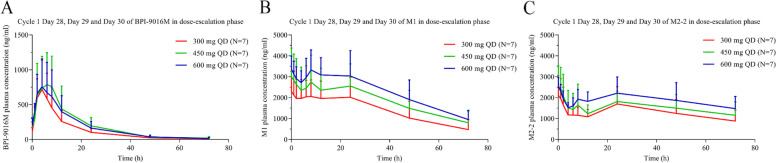
Mean plasma concentration–time profiles of BPI-9016M and its main metabolites (M1 and M2-2). **A:** Plasma concentration–time curve of BPI-9016M PK assessed in Cycle 1 Day 28 of dose-expansion phase at 648 h, 649 h, 650 h, 652 h, 654 h, 656 h, 660 h, 672 h, 696 h, 720 h after Cycle 1 first dose of BPI-9016M. **B:** Plasma concentration–time curve of M1 PK assessed in Cycle 1 Day 28 of dose-expansion phase at 648 h, 649 h, 650 h, 652 h, 654 h, 656 h, 660 h, 672 h, 696 h, 720 h after Cycle 1 first dose of BPI-9016M. **C:** Plasma concentration–time curve of M2-2 PK assessed in Cycle 1 Day 28 of dose-expansion phase at 648 h, 649 h, 650 h, 652 h, 654 h, 656 h, 660 h, 672 h, 696 h, 720 h after Cycle 1 first dose of BPI-9016M *QD* quaque die, *PK* pharmacokinetics

A total of 21 patients in Part A were included in PKAS. Thirteen patients in Part A were not included in PKAS, the reason included: one patient in the 300 mg QD cohort had protocol violation and no exact administration time of BPI-9016M; one patient in the 600 mg QD cohort discontinued over two-week treatment of BPI-9016M due to the poor compliance; and the sparse plasma PK data from other 11 patients in Part A (four patients in the 300 mg QD cohort, five patients in the 450 mg QD cohort, and two patients in the 600 mg QD cohort) were analyzed based on population PK analysis without PK modeling. Therefore, in this study, we only used the PK data of 21 patients to assess the PK profiles of BPI-9016M and its metabolites (M1 and M2-2). Following the Cycle 1 of BPI-9016M treatment, PK parameters of BPI-9016M, and its major metabolites M1 and M2-2 were summarized in Table 4 and Fig. 4. According to the results among the three dose cohorts (300 mg QD, 450 mg QD and 600 mg QD) in PKAS after multiple doses for 28 days, the median T_max,ss_ of BPI-9016M was 4.0 h, other parameters for BPI-9016M such as the mean CL_ss_/F ranged from 50.4 L/h to 83.2 L/h, the mean T_1/2_ ranged from 11.2 h to 12.7 h and the mean V_z,ss_/F ranged from 781.0 L to 1780.0 L. These PK parameters showed no clear difference to previous phase I results [20]. The mean T_1/2_ of M1 ranged from 19.8 h to 21.8 h. For M2-2, its mean T_1/2_ was not able to estimate in the 450 mg QD and 600 mg QD cohorts, and 21.3 h in the 300 mg QD cohort. The MP ratio calculated based on AUC_tau_ ranged from 6.0 to 9.1 for M1, and ranged from 4.1 to 5.7 for M2-2. Major PK parameters of BPI-9016M, M1 and M2-2 after treatment with multiple doses of BPI-9016M for 28 days in Cycle 1 based on population PK analysis were given in Supplementary Table 3. The mean trough concentration of BPI-9016M, M1 and M2-2 after treatment with multiple doses of BPI-9016M at Cycle 1 Day 8, Day 15 and Day 22 based on routine PK analysis were listed in Supplementary Table 4.

**Table 4:** Major PK parameters of BPI-9016M, M1 and M2-2 after treatment with multiple doses of BPI-9016M for 28 days in Part A

PK Parameters	300 mg QD (*n* = 7) Mean (SD)	450 mg QD (*n* = 7) Mean (SD)	600 mg QD (*n* = 7) Mean (SD)
**AUC**_**tau**_** (h × ng/mL)**			
BPI-9016M	7680.0 (4120.0)	11,000.0 (5970.0)	10,100.0 (5350.0)
M1	48,200.0 (20,500.0)	60,200.0 (25,100.0)	73,400.0 (19,600.0)
M2-2	32,400.0 (12,200.0)	38,200.0 (13,500.0)	46,300.0 (10,600.0)
**C**_**max,ss**_** (ng/mL)**			
BPI-9016M	794.0 (370.0)	932.0 (480.0)	788.0 (394.0)
M1	2510.0 (969.0)	3150.0 (1310.0)	3880.0 (863.0)
M2-2	2300.0 (940.0)	2610.0 (972.0)	2730.0 (344.0)
**C**_**min,ss**_** (ng/mL)**			
BPI-9016M	104.0 (49.0)	168.0 (127.0)	157.0 (89.6)
M1	1770.0 (910.0)	2100.0 (818.0)	2540.0 (601.0)
M2-2	1030.0 (342.0)	1150.0 (373.0)	1450.0 (269.0)
**T**_**max,ss**_** (h)**			
BPI-9016M	3.9 (2.0)	4.0 (1.0)	3.9 (2.0)
M1	0.0 (0.0)	1.1 (0.0)	1.0 (0.0)
M2-2	0.0 (0.0)	0.0 (0.0)	1.0 (0.0)
**T**_**1/2**_** (h)**			
BPI-9016M	11.2 (2.8)	11.8 (4.8)	12.7 (4.8)
M1	19.8 (3.6)	21.7 (3.0)	21.8 (2.9)
M2-2	21.3 (NE)	NE (NE)	NE (NE)
**C**_**trough**_** (ng/mL)**			
BPI-9016M	113.5 (55.0)	184.1 (131.2)	203.9 (99.2)
M1	2294.0 (965.6)	3002.9 (1370.5)	3372.9 (615.1)
M2-2	2112.4 (952.7)	2564.3 (966.6)	2544.3 (432.4)
**CL**_**ss**_**/F (L/h)**			
BPI-9016M	50.4 (25.6)	57.6 (39.5)	83.2 (58.9)
**V**_**z,ss**_**/F (L)**			
BPI-9016M	781.0 (357.0)	1050.0 (974.0)	1780.0 (2080.0)
**MP ratio**			
M1	7.0 (2.1)	6.0 (1.4)	9.1 (4.7)
M2-2	4.9 (2.2)	4.1 (2.0)	5.7 (3.2)

Data are mean (SD), unless otherwise stated. One patient in the 300 mg QD cohort had protocol violation and no exact administration time of BPI-9016M. One patient in the 600 mg QD cohort discontinued over two-week treatment of BPI-9016M due to the poor compliance. The sparse plasma PK data from other 11 patients were analyzed based on population PK analysis without PK modeling, therefore 21 patients were included in PKAS

*PK* pharmacokinetics, *AUC* area under the concentration-time curve, *AUC*_*tau*_ *AUC* over a dosing interval, *C*_*max,ss*_ maximum plasma concentration at steady state, *C*_*min,ss*_ minimum plasma concentration at steady state, *T*_*max,ss*_ time to maximum plasma concentration at steady state, *T*_*1/2*_ terminal time of half-life, *C*_*trough*_ trough concentration, *CL*_ss_*/F* overall body clearance at steady state for extravascular dosage, *V*_*z,ss*_*/F* total volume of drug distribution at steady state according to the terminal phase, *MP ratio* ratio of metabolites to parent drug calculated based on AUC_tau_, *QD* quaque die, *SD* standard deviation, *NE* not evaluable, *PKAS* pharmacokinetics analysis set

## Discussion

BPI-9016M is a novel small-molecule TKI that targets both c-MET and AXL, which inhibits tumor cell growth, migration and invasion in NSCLC. This phase Ib dose-expansion study in 38 c-MET overexpression or *MET* exon 14 skipping mutation patients with locally advanced or metastatic NSCLC showed that BPI-9016M was well-tolerated in each dose cohort, and exhibited potential antitumor activity in those with c-MET overexpression or *MET* exon 14 skipping mutation.

This study showed that 92.1% of patients had TRAEs, and 28.9% had grade ≥ 3 TRAEs. One (2.6%) patient discontinued the study treatment owing to TRAE. The most common TRAEs were elevated ALT (36.8%), elevated AST (28.9%), dysgeusia (23.7%), PPE (23.7%) and constipation (21.1%). No treatment-related deaths occurred. The phase II GEOMETRY mono-1 study evaluated capmatinib in *MET* exon 14-mutated or *MET*-amplified NSCLC patients. The most common TEAEs were peripheral edema (51%) and nausea (45%), and most of them were grade 1 or 2. A total of 39 (11%) patients discontinued the treatment due to TRAEs [14]. In the phase II VISION study of patients with NSCLC and *MET* exon 14 skipping mutation treated with tepotinib, 42 (28%) patients experienced grade ≥ 3 TEAEs, including peripheral edema (7%). In addition, 11% of patients discontinued tepotinib treatment due to TRAEs [13]. A phase II study of savolitinib in Chinese patients with NSCLC harboring *MET* exon 14 skipping mutation showed that the incidence of grade ≥ 3 TRAEs was 46%, with the most common being increased AST (13%), increased ALT (10%) and peripheral oedema (9%) [15]. The safety profile of BPI-9016M was numerically similar with other MET TKIs in previous studies [13–15], and no new safety signals were identified.

The GEOMETRY mono-1 study showed that the ORR with capmatinib was 41% in patients with previously treated *MET* exon 14-mutated NSCLC and 68% in treatment-naïve patients. Further analyses for patients with *MET* amplification showed limited efficacy in those with gene copy number < 10 (ORR: 7–12%), but favorable efficacy in those with gene copy number ≥ 10 (ORR: 29% in patients with previously treated NSCLC, and 40% in treatment-naïve patients). Seven of 13 patients who had brain metastases at baseline achieved intracranial response, including four CR [14]. In the phase II study of tepotinib, the ORR was 55% (95% CI 23–83%) in 11 NSCLC patients harboring *MET* exon 14 skipping mutation with brain metastases [13]. Another phase II study of savolitinib, the DCR was 93.3% (95% CI 68.1–99.8%) in the 15 *MET* exon 14-mutated NSCLC patients with brain metastases [15]. In a retrospective real-world study, capmatinib yielded an ORR of 58% and a median PFS of 9.5 months in *MET* exon 14-mutated NSCLC [22]. The VISION study of tepotinib exhibited an ORR of 56% (95% CI 45–66%) in patients with locally advanced or metastatic NSCLC and *MET* exon 14 skipping mutation, regardless of previously treated or untreated NSCLC investigator assessed [13]. The phase II study of savolitinib in Chinese patients with NSCLC harboring *MET* exon 14 skipping mutation showed an ORR of 42.9% (95% CI 31.1–55.3%) in FAS [15]. The phase II AcSé study of crizotinib in patients with *MET*- or *ROS1*-positive NSCLC showed that the ORR was 16% in patients with *MET* copy number ≥ 6, and 10.7% in patients with *MET* mutation [23]. In this study, the ORR of BPI-9016M was only 2.6% (1/38, 95% CI 0.1–13.8%) in FAS, the ORR was 2.9% (1/34, 95% CI 0.07–15.33%) and 0% in Part A and Part B, respectively. One PR patient was observed at a dose of 600 mg QD in Part A. Though this patient suffered from TRAE, the treatment was not discontinued and PR was achieved. Part A of this study enrolled patients with c-MET overexpression (IHC staining score ≥ 2 +), without screening of *MET* exon 14 skipping mutation or *MET* amplification. This was inconsistent with other studies which only enrolled patients with *MET* exon 14 skipping mutation or *MET* amplification. The GEOMETRY mono-1 study showed that patients with *MET* exon 14 skipping mutation seemed to have better treatment response and survival than those with *MET* amplification [14]. This indicated that patients with *MET* exon 14 skipping mutation might be a better target population, which also supported our further study in Part B. However, due to the limited sample size and short follow-up duration, the activity of BPI-9016M in patients with both c-MET overexpression and *MET* exon 14 skipping mutation still needs further investigation.

The c-MET is overexpressed in approximately 35–72% of NSCLC and was determined by IHC [10, 11]. C-MET overexpression induces ligand-independent phosphorylation that influences downstream signaling pathways, which is linked to poor survival [11]. An IHC score 3 + was observed in 11 of 76 patients with *MET*-positive solid tumors in the phase I study of capmatinib [17]. A total of 48 patients with c-MET overexpression (H-score of ≥ 150) were enrolled into the phase I study of telisotuzumab vedotin in patients with advanced solid tumors [18]. Nevertheless, MET TKIs have not yet been studied extensively in NSCLC patients with c-MET overexpression. In this study, c-MET overexpression was defined as IHC score ≥ 2 + , which was inconsistent with previous studies [17]. Further study is needed to determine the threshold for IHC scores indicating c-MET overexpression.

Given the above-mentioned limitations, we suggest some valuable points for future studies. First, monotherapy of BPI-9016M targeting patients with c-MET overexpression (IHC staining score 3 +), *MET* amplification or *MET* exon 14 skipping mutation would probably present promising results, as subgroup analyses of previous study has suggested that patients with c-MET overexpression of IHC 3 + owned good sensitivity to such therapy [24]. Then, combination therapy containing BPI-9016M and EGFR TKIs for patients with *EGFR* sensitizing mutation, or patients with c-MET dysregulation and acquired resistance to EGFR TKIs worth further investigation. Moreover, given the high programmed cell death-ligand 1 (PD-L1) expression level reported in patients with c-MET overexpression or *MET* amplification [25], BPI-9016M combined with immunotherapy for this patient population would also be a potential focus. However, the expression of PD-L1 was not detected at baseline in this study, which should be further evaluated.

In summary of this study, BPI-9016M showed manageable safety profile in c-MET overexpression or *MET* exon 14 skipping mutation patients with locally advanced or metastatic NSCLC, but showed limited efficacy. Patients with *MET* exon 14 skipping mutation seemed to have relatively preferable antitumor activity. Further exploration of BPI-9016M will be conducted in a larger sample size with both c-MET overexpression and *MET* exon 14 skipping mutation.

## Supplementary Information

**Additional file 1: Supplementary Table 1. **Dose adjustment criteria. **Supplementary Table 2. **TRAEs of BPI-9016M occurring in SS. **Supplementary Table 3. **Major PK parameters of BPI-9016M, M1and M2-2 after treatment with multiple doses of BPI-9016M for 28 days in Cycle 1 based on population PK analysis. **Supplementary Table 4. **Mean C_trough_ of BPI-9016M, M1 and M2-2 after treatment with multiple doses of BPI-9016M at Cycle 1 Day 8, Day 15 and Day 22 based on routine PK analysis.

## Data Availability

Data generated and analysed in this study are on file with Betta Pharmaceuticals Co., Ltd, Hangzhou, China, and are not publicly available according to the company. The data (such as efficacy and safety) produced during the trial are available from the corresponding authors upon reasonable request.
