# Supplementary material for: Safety, efficacy and pharmacokinetics of BPI-9016M in c-MET overexpression or MET exon 14 skipping mutation patients with locally advanced or metastatic non-small-cell lung cancer: a phase Ib study
Source: BMC Cancer. 2023 Apr 11;23:331. doi: 10.1186/s12885-022-10500-y (PMC10088252; doi:10.1186/s12885-022-10500-y)
Supplement: Supplementary file 1 — Additional file 1: Supplementary Table 1. Dose adjustment criteria. Supplementary Table 2. TRAEs of BPI-9016M occurring in SS. Supplementary Table 3. Major PK parameters of BPI-9016M, M1and M2-2 after treatment with multiple doses of BPI-9016M for 28 days in Cycle 1 based on population PK analysis. Supplementary Table 4. Mean Ctrough of BPI-9016M, M1 and M2-2 after treatment with multiple doses of BPI-9016M at Cycle 1 Day 8, Day 15 and Day 22 based on routine PK analysis. [file 12885_2022_10500_MOESM1_ESM.pdf]

Supplementary Table 1 Dose adjustment criteria

Toxicity		Measure	Dose adjustment
Grade 4 neutropenia or grade 3 neutropenia accompanied with fever (≥ 38.5 ˚C) for ≥ 5 days	First occurrence	Interrupt the treatment, and recover the neutrophil count to ≥ 1.5 × 10^9/L and body temperature to < 38˚C	Reduce the dose to the last level (by at least 25%)
	Second occurrence	Discontinue the treatment, and recover the neutrophil count to ≥ 1.5 × 10^9/L and body temperature to < 38˚C	Terminate the study[a]
Grade ≥ 3 thrombopenia	Platelet count < 25 × 10^9/L at first occurrence	Discontinue the treatment, and recover the platelet count to ≥ 80 × 10^9/L	Terminate the study[a]
	Platelet count ≥ 25 × 10^9/L and < 50×10^9/L at first occurrence	Interrupt the treatment, and recover the platelet count to ≥ 80 × 10^9/L	Reduce the dose to the last level (by at least 25%)
	Platelet count < 50 × 10^9/L at second occurrence	Discontinue the treatment, and recover the platelet count to ≥ 80 × 10^9/L	Terminate the study[a]
Any grade nonhemolytic anemia	First occurrence	Interrupt the treatment, and control the anemia with blood transfusion	Reduce the dose to the last level (by at least 25%)
	Second occurrence	Discontinue the treatment, and control the anemia with blood transfusion	Terminate the study[a]

[a]Treatment of BPI-9016M should be discontinued unless the investigator and sponsor agree to continue the treatment BPI-9016M at a reduced dose level, which conforms to the patient's maximum

benefit.

Supplementary Table 2 TRAEs of BPI-9016M occurring in SS

TRAEs	Part A						Part B		Total (n=38) n (%)	
	300 mg QD (n=12) n (%)		450 mg QD (n=12) n (%)		600 mg QD (n=10) n (%)		400 mg BID (n=4) n (%)			
	Any grade	Grade 3-4	Any grade	Grade 3-4	Any grade	Grade 3-4	Any grade	Grade 3-4	Any grade	Grade 3-4
Elevated ALT	3 (25.0)	0	5 (41.7)	0	3 (30.0)	0	3 (75.0)	0	14 (36.8)	0
Elevated AST	2 (16.7)	0	5 (41.7)	0	1 (10.0)	0	3 (75.0)	0	11 (28.9)	0
Dysgeusia	1 (8.3)	0	3 (25.0)	0	5 (50.0)	1 (10.0)	0	0	9 (23.7)	1 (2.6)
PPE	1 (8.3)	0	3 (25.0)	1 (8.3)	1 (10.0)	0	4 (100.0)	3 (75.0)	9 (23.7)	4 (10.5)
Constipation	2 (16.7)	0	5 (41.7)	0	1 (10.0)	0	0	0	8 (21.1)	0
Elevated blood creatinine	3 (25.0)	0	2 (16.7)	0	1 (10.0)	0	1 (25.0)	0	7 (18.4)	0
Decreased Ccr	2 (16.7)	0	2 (16.7)	0	2 (20.0)	0	0	0	6 (15.8)	0
Asthenia	0	0	2 (16.7)	0	3 (30.0)	1 (10.0)	0	0	5 (13.2)	1 (2.6)
Decreased appetite	2 (16.7)	0	2 (16.7)	0	1 (10.0)	1 (10.0)	0	0	5 (13.2)	1 (2.6)
Proteinuria	0	0	3 (25.0)	1 (8.3)	1 (10.0)	0	0	0	4 (10.5)	1 (2.6)
Hypertension	1 (8.3)	0	0	0	2 (20.0)	1 (10.0)	1 (25.0)	0	4 (10.5)	1 (2.6)
Elevated bilirubin	0	0	0	0	2 (20.0)	0	1 (25.0)	0	3 (7.9)	0
Elevated creatine phosphokinase	0	0	1 (8.3)	0	0	0	2 (50.0)	0	3 (7.9)	0
Diarrhoea	1 (8.3)	0	1 (8.3)	0	1 (10.0)	0	0	0	3 (7.9)	0
Dry mouth	1 (8.3)	0	2 (16.7)	0	0	0	0	0	3 (7.9)	0
Haematuria	0	0	3 (25.0)	0	0	0	0	0	3 (7.9)	0
Nausea	3 (25.0)	0	0	0	0	0	0	0	3 (7.9)	0
Thrombocytopenia	0	0	0	0	1 (10.0)	1 (10.0)	2 (50.0)	1 (25.0)	3 (7.9)	2 (5.3)
Urinary tract infection	0	0	1 (8.3)	0	2 (20.0)	0	0	0	3 (7.9)	0
Ventricular arrhythmia	1 (8.3)	0	1 (8.3)	0	1 (10.0)	0	0	0	3 (7.9)	0

Leukopenia	0	0	1 (8.3)	0	1 (10.0)	0	1 (25.0)	0	3 (7.9)	0
Activated partial thromboplastin time prolonged	1 (8.3)	0	1 (8.3)	0	0	0	0	0	2 (5.3)	0
Anaemia	0	0	1 (8.3)	1 (8.3)	1 (10.0)	0	0	0	2 (5.3)	1 (2.6)
Hypoaesthesia oral	0	0	2 (16.7)	0	0	0	0	0	2 (5.3)	0
Neuritis	0	0	0	0	2 (20.0)	0	0	0	2 (5.3)	0
Occult blood	0	0	1 (8.3)	0	1 (10.0)	0	0	0	2 (5.3)	0
Oedema peripheral	0	0	1 (8.3)	0	1 (10.0)	0	0	0	2 (5.3)	0
Vomiting	0	0	1 (8.3)	0	0	0	1 (25.0)	0	2 (5.3)	0
Accessory nerve disorder	1 (8.3)	0	0	0	0	0	0	0	1 (2.6)	0
Elevated alpha-HBDH	0	0	0	0	0	0	1 (25.0)	0	1 (2.6)	0
Anaesthesia oral	0	0	0	0	1 (10.0)	0	0	0	1 (2.6)	0
Arrhythmia	0	0	0	0	1 (10.0)	0	0	0	1 (2.6)	0
Arthralgia	0	0	1 (8.3)	0	0	0	0	0	1 (2.6)	0
Blister	0	0	1 (8.3)	0	0	0	0	0	1 (2.6)	0
Elevated LDH	0	0	0	0	0	0	1 (25.0)	0	1 (2.6)	0
Elevated serum urea	1 (8.3)	0	0	0	0	0	0	0	1 (2.6)	0
Hypotension	1 (8.3)	0	0	0	0	0	0	0	1 (2.6)	0
Chest discomfort	1 (8.3)	0	0	0	0	0	0	0	1 (2.6)	0
Chronic kidney disease	0	0	1 (8.3)	0	0	0	0	0	1 (2.6)	0
Cough	0	0	0	0	1 (10.0)	0	0	0	1 (2.6)	0
Dizziness	1 (8.3)	0	0	0	0	0	0	0	1 (2.6)	0
Dyskinesia	0	0	0	0	1 (10.0)	1 (10.0)	0	0	1 (2.6)	1 (2.6)
Dysphonia	0	0	0	0	1 (10.0)	0	0	0	1 (2.6)	0
Dyspnoea	0	0	0	0	1 (10.0)	1 (10.0)	0	0	1 (2.6)	1 (2.6)

Electrocardiogram QT prolonged	1 (8.3)	0	0	0	0	0	0	0	1 (2.6)	0
Elevated D dimer	0	0	0	0	1 (10.0)	0	0	0	1 (2.6)	0
Elevated GGT	0	0	0	0	0	0	1 (25.0)	0	1 (2.6)	0
Genital rash	0	0	0	0	1 (10.0)	0	0	0	1 (2.6)	0
Headache	1 (8.3)	0	0	0	0	0	0	0	1 (2.6)	0
Hyperhidrosis	0	0	1 (8.3)	0	0	0	0	0	1 (2.6)	0
Hyperlipidaemia	0	0	0	0	0	0	1 (25.0)	0	1 (2.6)	0
Hypocalcaemia	0	0	0	0	1 (10.0)	0	0	0	1 (2.6)	0
Insomnia	1 (8.3)	0	0	0	0	0	0	0	1 (2.6)	0
Onycholysis	0	0	1 (8.3)	0	0	0	0	0	1 (2.6)	0
Oropharyngeal pain	0	0	0	0	1 (10.0)	1 (10.0)	0	0	1 (2.6)	1 (2.6)
Paraesthesia	0	0	1 (8.3)	0	0	0	0	0	1 (2.6)	0
Pruritus	0	0	1 (8.3)	0	0	0	0	0	1 (2.6)	0
Rash maculo-papular	1 (8.3)	0	0	0	0	0	0	0	1 (2.6)	0
Sinus tachycardia	0	0	0	0	1 (10.0)	0	0	0	1 (2.6)	0
Skin fissures	0	0	0	0	1 (10.0)	1 (10.0)	0	0	1 (2.6)	1 (2.6)
Supraventricular extrasystoles	0	0	0	0	1 (10.0)	0	0	0	1 (2.6)	0
Elevated troponin	0	0	1 (8.3)	0	0	0	0	0	1 (2.6)	0
Upper respiratory tract infection	1 (8.3)	0	0	0	0	0	0	0	1 (2.6)	0
Urinary retention	0	0	0	0	1 (10.0)	0	0	0	1 (2.6)	0
Ventricular extrasystoles	1 (8.3)	0	0	0	0	0	0	0	1 (2.6)	0
Weight loss	0	0	0	0	1 (10.0)	0	0	0	1 (2.6)	0

Data are n (%). One patient in the 450 mg QD cohort suffered an AE of pulmonary infection leading to death, which was deemed unrelated to BPI-9016M.

TRAEs treatment-related adverse events, *SS* safety set, *ALT* alanine aminotransferase, *AST* aspartate aminotransferase, *PPE* palmar-plantar erythrodysesthesia, *Ccr* creatinine clearance, *HBDH* hydroxybutyrate dehydrogenase, *LDH* lactate dehydrogenase, *GGT* gamma-glutamyltransferase, *QD* quaque die, *BID* bis in die

Supplementary Table 3 Major PK parameters of BPI-9016M, M1 and M2-2 after treatment with multiple doses of BPI-9016M for 28 days in Cycle 1 based on population PK analysis

PK parameter	AUC_{tau} (h×ng/mL)	$C_{max,ss}$ (ng/mL)	$C_{min,ss}$ (ng/mL)	$T_{max,ss}$ (h)	$T_{1/2}$ (h)	C_{trough} (ng/mL)	CL_{ss}/F (L/h)	$V_{z,ss}$/F (L)
**300 mg QD (n=4)**	**Mean (SD)**	**Mean (SD)**	**Mean (SD)**	**Mean (SD)**	**Mean (SD)**	**Mean (SD)**	**Mean (SD)**	**Mean (SD)**
BPI-9016M	7420.0 (3120.0)	601.0 (189.0)	89.1 (56.3)	4.0 (2.0)	9.1 (1.7)	94.1 (79.0)	46.2 (18.7)	685.0 (196.0)
M1	40200.0 (22000.0)	2020.0 (1090.0)	1560.0 (969.0)	5.5 (0.0)	29.0 (0.1)	4018.2 (2405.0)	NE	NE
M2-2	22600.0 (7450.0)	1290.0 (351.0)	775.0 (291.0)	2.5 (0.0)	NE	1355.0 (1336.4)	NE	NE
**450 mg QD (n=5)**	**Mean (SD)**	**Mean (SD)**	**Mean (SD)**	**Mean (SD)**	**Mean (SD)**	**Mean (SD)**	**Mean (SD)**	**Mean (SD)**
BPI-9016M	6350.0 (2710.0)	470.0 (83.3)	151.0 (77.5)	5.1 (2.0)	10.3 (NE)	167.0 (100.5)	77.9 (33.2)	1500.0 (NE)
M1	51600.0 (28600.0)	3780 (2170.0)	2660.0 (1400.0)	0.0 (0.0)	NE	2964.2 (1808.6)	NE	NE
M2-2	35900.0 (5180.0)	2360.0 (744.0)	1460.0 (476.0)	0.0 (0.0)	NE	2880.0 (1216.2)	NE	NE
**600 mg QD (n=2)**	**Mean (SD)**	**Mean (SD)**	**Mean (SD)**	**Mean (SD)**	**Mean (SD)**	**Mean (SD)**	**Mean (SD)**	**Mean (SD)**
BPI-9016M	6980.0 (NE)	758.0 (NE)	80.3 (NE)	3.0 (NE)	9.6 (NE)	201.1 (166.4)	86.0 (NE)	1180.0 (NE)
M1	40900.0 (NE)	1810.0 (NE)	1610.0 (NE)	8.0 (NE)	NE	3420.0 (999.1)	NE	NE
M2-2	27200.0 (NE)	1520.0 (NE)	956.0 (NE)	0.0 (NE)	NE	912.0 (661.9)	NE	NE

Data are mean (SD), unless otherwise stated.

PK pharmacokinetics, *QD* quaque die, *AUC* area under the concentration-time curve, AUC_{tau} AUC over a dosing interval, $C_{max,ss}$ maximum plasma concentration at steady state, $C_{min,ss}$ minimum plasma concentration at steady state, $T_{max,ss}$ time to maximum plasma concentration at steady state, $T_{1/2}$ terminal time of half-life, C_{trough} trough concentration, CL_{ss}/F overall body clearance at steady state for extravascular dosage, $V_{z,ss}/F$ total volume of drug distribution at steady state according to the terminal phase, *SD* standard deviation, *NE* not evaluable

Supplementary Table 4 Mean C_{trough} of BPI-9016M, M1 and M2-2 after treatment with multiple doses of BPI-9016M at Cycle 1 Day 8, Day 15 and Day 22 based on routine PK analysis

C_{trough} (ng/mL)	300 mg QD (n=7)	450 mg QD (n=7)	600 mg QD (n=7)
**Cycle 1 Day 8**	**Mean (SD)**	**Mean (SD)**	**Mean (SD)**
BPI-9016M	163.0 (241.5)	178.1 (128.0)	165.6 (66.6)
M1	1508.6 (291.5)	3349.7 (1605.1)	3260.0 (582.3)
M2-2	1263.6 (346.8)	2894.1 (1279.3)	2264.3 (551.7)
**Cycle 1 Day 15**	**Mean (SD)**	**Mean (SD)**	**Mean (SD)**
BPI-9016M	161.5 (126.3)	216.5 (111.8)	188.7 (67.3)
M1	1956.4 (983.2)	3628.6 (1418.2)	3651.4 (1481.6)
M2-2	1529.6 (552.8)	3382.9 (1077.3)	3032.9 (1305.9)
**Cycle 1 Day 22**	**Mean (SD)**	**Mean (SD)**	**Mean (SD)**
BPI-9016M	124.2 (126.2)	236.3 (83.4)	164.1 (65.7)
M1	2276.6 (1742.0)	3712.9 (644.7)	2982.9 (570.9)
M2-2	1684.3 (725.5)	2730.0 (600.7)	2578.6 (772.4)

Data are mean (SD), unless otherwise stated. A single blood collection was performed at Cycle 1 Day 8 (192 h), Day 15 (360 h) and Day 22 (528 h) for routine PK analysis, respectively.

C_{trough} trough concentration, *PK* pharmacokinetics, *QD* quaque die, *SD* standard deviation
